# Drug Treatment Attenuates Retinal Ganglion Cell Death by Inhibiting Collapsin Response Mediator Protein 2 Phosphorylation in Mouse Models of Normal Tension Glaucoma

**DOI:** 10.1007/s12017-024-08778-1

**Published:** 2024-04-15

**Authors:** Yuebing Wang, Musukha Mala Brahma, Kazuya Takahashi, Alessandra Nolia Blanco Hernandez, Koki Ichikawa, Syuntaro Minami, Yoshio Goshima, Takayuki Harada, Toshio Ohshima

**Affiliations:** 1 Department of Life Science and Medical Bioscience, Waseda University, Shinjuku-ku, Tokyo, 162-8480 Japan; 2 Nara Medical University, Kashihara City, Nara 634-8521 Japan; 3 Department of Molecular Pharmacology and Neurobiology, Yokohama City University Graduate School of Medicine, Yokohama, 236-0004 Japan; 4 Visual Research Project, Tokyo Metropolitan Institute of Medical Science, Tokyo, 156-8506 Japan; 5 Laboratory for Molecular Brain Science, Department of Life Science and Medical Bioscience, Waseda University, 2-2 Wakamatsu-cho, Shinjuku-ku, Tokyo, 162-8480 Japan

**Keywords:** CRMP2, Normal tension glaucoma, Retinal ganglion cell, Phosphorylation

## Abstract

Normal tension glaucoma (NTG) is a progressive neurodegenerative disease in glaucoma families. Typical glaucoma develops because of increased intraocular pressure (IOP), whereas NTG develops despite normal IOP. As a subtype of open-angle glaucoma, NTG is characterized by retinal ganglion cell (RGC) degeneration, gradual loss of axons, and injury to the optic nerve. The relationship between glutamate excitotoxicity and oxidative stress has elicited great interest in NTG studies. We recently reported that suppressing collapsin response mediator protein 2 (CRMP2) phosphorylation in S522A CRMP2 mutant (CRMP2 KIKI) mice inhibited RGC death in NTG mouse models. This study evaluated the impact of the natural compounds huperzine A (HupA) and naringenin (NAR), which have therapeutic effects against glutamate excitotoxicity and oxidative stress, on inhibiting CMRP2 phosphorylation in mice intravitreally injected with *N*-methyl-d-aspartate (NMDA) and GLAST mutant mice. Results of the study demonstrated that HupA and NAR significantly reduced RGC degeneration and thinning of the inner retinal layer, and inhibited the elevated CRMP2 phosphorylation. These treatments protected against glutamate excitotoxicity and suppressed oxidative stress, which could provide insight into developing new effective therapeutic strategies for NTG.

## Introduction

Glaucoma is a group of retinal neurodegenerative diseases characterized by progressive death of the retinal ganglion cells (RGCs) and their axons, reduction of inner retinal layer (IRL) thickness, and optic nerve (ON) degeneration, leading to visual impairments. It is the leading cause of unalterable blindness globally, with increasing incidence due to the involvement of age-related factors (Quigley, 2006; Tham et al., 2014; Weinreb & Khaw, 2004). Glaucoma is generally considered a condition with elevated intraocular pressure (IOP) of > 21 mmHg. Normal tension glaucoma (NTG) is a type of primary open-angle glaucoma that occurs despite an IOP level within an average range, supporting that other IOP-independent disease factors also contribute to the pathophysiology of glaucoma, including excitotoxicity, oxidative stress, and mitochondrial dysfunction (Kikuchi et al., 2020). Moreover, decreased glutamine transporter and the antioxidant glutathione have been detected in the retinas and plasma of patients with glaucoma (Gherghel et al., 2005; Naskar et al., 2000). The molecular mechanisms of RGC apoptosis are frequently studied using animal models of *N*-methyl-d-aspartate (NMDA)-induced excitotoxic damage to RGC (Seitz et al., 2010). GLAST expressed in Müller cells is vital for maintaining the extracellular glutamate concentration below the neurotoxic level and the glutathione levels within a normal range in the mammalian retina (Harada et al., 2007). GLAST mutant mice exhibited RGC loss and ON degeneration with normal IOP (Dong et al., 2016; Sano et al., 2019; Tanaka-Gonome et al., 2020).

Collapsin response mediator protein 2 (CRMP2) is an intracellular protein expressed in neurons that mediates signals for semaphorins and neurotrophins. It is involved in axonal guidance, dendritic spine development, and synaptic plasticity through phosphorylation (Yamashita et al., 2012). CRMP2 binds to tubulin dimers and facilitates axonal elongation. This function is terminated in phosphorylated CRMP2 and acts as an intracellular signalling mediator in inhibiting axonal guidance via semaphorin 3A (Sema3A) (Nakamura et al., 2020). CRMP2 is phosphorylated by cyclin-dependent kinase 5 (Cdk5) at Ser522 in Sema3A signalling, followed by further phosphorylation by glycogen synthase kinase-3β (GSK-3β) (Yamashita et al., 2012). The phosphorylation of CRMP2 (pCRMP2) is linked to several neurological dysfunctions, such as Alzheimer’s disease (AD) and spinal injury (Cole et al., 2007; Menon et al., 2011; Petratos et al., 2012; Yoshida et al., 1998; Zhao et al., 2020). S522A CRMP2 mutant (CRMP2KI/KI) mice do not show RGC loss as observed in the wild-type mice with ON axon degeneration after injury (Kondo et al., 2019). We recently reported that RGC death was inhibited due to the suppression of CRMP2 phosphorylation in the NTG CRMP2KI/KI mice (Brahma et al., 2022).

(-)-Huperzine A is an alkaloid isolated from the herb *Huperzia serrata*. It has been used for centuries in Chinese folk medicine as a remedy for inflammation, schizophrenia, and memory loss (Friedli & Inestrosa, 2021). Previous studies demonstrate that Huperzine A is involved in pharmacological activities, including inhibiting acetylcholinesterase (AChE) and attenuating the toxicity of β-amyloid (Herzon & Tun, 2012). The overexcitation of the *N*-methyl-d-aspartate (NMAD) receptor, a glutamate receptor, is involved in several neurodegenerative diseases, such as AD and epilepsy. HupA also protects against glutamate-induced neurotoxicity (Zhang & Tang, 2006) and inhibits oxidative stress (Herzon & Tun, 2012). These all highly associated with NTG development. Also, HupA was found to suppress CRMP2 phosphorylation in a previous study (Zhao et al., 2020), which led to the consequent consideration of its effect on RGC apoptosis in the NTG mouse model.

Naringenin (2,3-dihydro-5,7-dihydroxy-2-(4-hydroxyphenyl)-4*H*-1 benzopyran-4-1) is a natural multitarget flavonoid predominantly found in citrus fruits, such as grapefruits, oranges, and lemons, as well as in a variety of other fruits and herbs. In different experimental neurodegenerative models, NAR showed promising therapeutic effects by targeting multiple targets and signalling pathways on many diseases through its activities, such as neuroprotection, antidiabetic effects in diabetic retinopathy, and antioxidant and anti-inflammatory activities (Al-Dosari et al., 2017). NAR induces conformational changes in CRMP2 and can disrupt target phosphorylation sites within the secondary structure of the C-terminal tail of CRMP2 (Lawal et al., 2018; Yang et al., 2017). NAR has a positive effect on ocular ischemia disease. In a study of glaucoma using the ocular hypertension model and ischemia/reperfusion model, NAR demonstrated amelioration of RGC death (Kara et al., 2014), which gives a clue to investigate the effect of NAR suppress CRMP2 phosphorylation in NTG mouse model.

NTG is commonly neglected since its symptoms are difficult to identify in the early stages. The severe consequences of retinal degeneration and RGC loss should not be overlooked. One of the long-term objectives of NTG research is to discover efficient neuroprotective agents. This study aimed to examine the roles of HupA and NAR in NTG pathology using two mouse models: NMDA-injected mice and GLAST mutant mice, which could help develop effective new therapies for NTG.

## Materials and Methods

### Experimental Animals

Animal experiments were approved by the Institutional Animal Care Use Committee of WASEDA University. Mice were maintained in 12-h dark and 12-h light nursery conditions with free access to food and water. As previously described (Sano et al., 2019; Tanaka-Gonome et al., 2020), GLAST mutant mice were generated in C57BL6 background and genotyped. Based on the previous study (Yamashita et al., 2012), the wild-type and CRMP2 KI/KI mice were created, and a hybrid background of 129 Sv C57BL/6J mice was maintained. GLAST+/− and CRMP2KI/KI mice were generated by crossing GLAST mutant and CRMP2KI/KI mice and genotyped (Brahma et al., 2022) All mice in this study were male.

### Intraocular Injection

Mice (10–16 weeks) were anesthetized with diethyl ether and intravitreally injected 20 nmol NMDA in 2 µL of PBS with a 33-gauge needle connected to a 10 µL Hamilton syringe to induce in vivo glutamate excitotoxicity in a glaucoma model. The injection needle was inserted approximately 1 mm behind the corneal limbus. NMDA was injected into the right eye, and PBS was injected into the left eye as a control.

### Treatment Strategies

HupA (Chemodex) and NAR (TCI, Japan) were orally administered to mice by mixing the powder with regular food. The mice were fed 0.7 mg/kg of HupA and 200 mg/kg of NAR daily. The mice were fed HupA and NAR 1 day before intraocular injection for the NMDA-injected experimental model. The feeding duration varied based on different experiments, as explained in the results section. For the GLAST mutant experimental model, GLAST+/− mice were fed the same amount of HupA and NAR administered in mice from 3-week-old until the end of 5- and 10-week-old. Mice in two age groups were analyzed using hematoxylin and eosin (H&E) staining (both 5- and 10-week-old mice) and immunostaining (10-week-old mice only).

### Histological Analysis-Immunohistochemistry

For flat-mount retina samples, mice were intracardially perfused with 4% paraformaldehyde (PFA) in 0.1% PBS (pH 7.4). The eyes were isolated from the eyecup of mice, enucleated, and dissected. Each retina was given four evenly spaced slits so that it could be located flat on slides. Retinal samples were immunostained as previously described (Kondo et al., 2019). Primary antibodies included anti-NeuN antibody (1:500, mouse monoclonal antibody MAB377, Millipore), which distinguish RGC by position and size in flat-mount retina, anti-pCRMP2 (S522) antibody (1:500, rabbit polyclonal antibody ab193226, Abcam), which labels phosphorylated CRMP2 at S522, and anti-RBPMS antibody (1:500, rabbit polyclonal antibody PA5-31231, Invitrogen, CA, USA), which is a marker of RGCs. Images of the immunostained retinal samples were captured and analyzed via a confocal microscope (FV1000, Olympus) and ImageJ. Multiple images of each section of the retina’s slit were captured at distances of 0.5 mm, 1.0 mm, and 1.5 mm from the optic disc. 12 confocal images are taken per retina, horizontal and vertical results were averaged for analysis.

For cryosection samples, the enucleated eyes were post-fixed for 1–2 h in a 4% glutaraldehyde solution, as described previously (Tanaka-Gonome et al., 2020). The eyes were then dissected, and the retina was placed in PBS at 4 °C (6–12 h). PBS was then replaced with 10% sucrose, and retina samples were incubated overnight at 4 °C and again transferred to 20% sucrose for 6–12 h at the same temperature. Retina samples were embedded in a 2:1 ratio of Tissue-Tek optimal cutting temperature compound and 20% sucrose in PBS to prepare a frozen block. Cryostat sections of the retina, 14 µm in thickness, were cut through the optic nerve and used for immunostaining. The frozen sections were immunostained overnight using an anti-RBPMS antibody (1:500, rabbit polyclonal antibody PA5-31231, Invitrogen, CA) and anti-4-hydroxynonenal (4-HNE) antibody (1:500, mouse monoclonal antibody, R&D systems), a marker for oxidative stress, followed by incubation with Alexa Fluor secondary antibodies at room temperature for 2 h, following several washes in PBS with 0.01% Triton-X100. A confocal microscope (FV1000, Olympus) was used to capture and analyze images of the samples. Three images of each sample captured at distances of 0.5 mm, 1.0 mm, and 1.5 mm from the optic disc were averaged for analysis. The intensities of 4-HNE staining in the ganglion cell layer of the mice were measured using ImageJ.

### Histological Analysis-H&E Staining

Diethyl ether was used to anesthetize mice before post-fixation in Zamboni’s fixative (0.1 M phosphate buffer, 15% picric acid, and 2% PFA), which was then used to post-fixate the mice for H&E staining. The samples were then embedded in a tissue processor (HistoCore PEARL, Leica) for 24 h. The embedding process was 70% EtOH, 80% EtOH, 90% EtOH for 2 h each, 100% EtOH for 4 h, xylene for 8 h, and paraffin for 6 h finally embedded in paraffin. The retinas were longitudinally sectioned (7 µm) through the optic nerve. H&E staining was performed as previously described (Brahma et al., 2022). H&E-stained retinas were imaged under a DP71 microscope (Olympus). The RGCs were counted from the ON head to the Ora serrata. The number of cells in the ganglion cell layer (GCL) and the thickness of the IRL (from the internal limiting membrane to the inner boundary of outer nuclear layer) were measured (Sano et al., 2019). Moreover, IRL thickness was measured in the areas 0.5 mm and 1 mm from the ON head.

### Statistical Analysis

All data were presented as the mean ± standard error of the mean. The statistical differences in the two-group analysis were compared using Student’s *t*-tests with unpaired two-tailed testing. A one-way analysis of variance (ANOVA) followed by Tukey’s post-hoc test was used for performing a statistical comparison for more than two groups. GraphPad Prism software version 8.3.0 was used for statistical analyses.

## Results

### Drug Treatment Inhibits Elevated CRMP2 Phosphorylation in the Retina of the NMDA-Administered Model

**Fig. 1:**
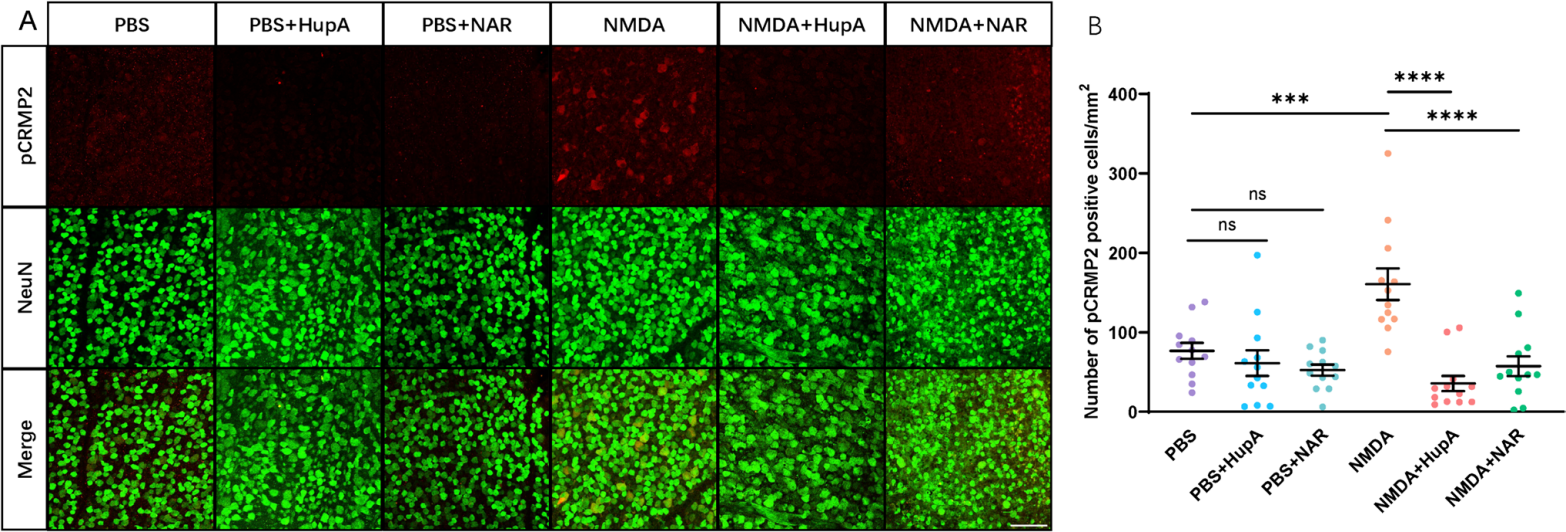
Drug treatments suppressed elevated pCRMP2 (S522) in the retina of mice with *N*-methyl-d-aspartate administration (10–16 weeks). HupA and NAR suppress the elevation of CRMP2 S522 phosphorylation 3 h after the administration of NMDA. NMDA of 20 nmol/2 μL was injected into the left side of the retina, and the same volume of PBS was injected into the right side of the retina as the control. **A** Immunostaining images of flat-mount mouse retina are shown. Scale bar, 100 μm. In the retinas of drug-treated mice, lower levels of phosphorylated CRMP2 S522 were detected by immunostaining with anti-NeuN and anti-pCRMP2 (S522) antibodies (NeuN: a marker of retinal ganglionic cells). **B** Quantitative analysis of pCRMP2-positive cells. Data are presented as the mean ± standard error of the mean (SEM) (*n* = 6) for all groups. ***p < 0.001; ****p < 0.0001; *ns* not significant

Our previous studies demonstrated that CRMP2 phosphorylation increased 3 h after NMDA administration (Brahma et al., 2022). Therefore, the pCRMP2 signal was detected in the flat-mount retina of the NMDA-administered mice with drug treatment (Fig. 1A, B). The wild-type mice were fed HupA and NAR with regular food powder 1 day before NMDA administration. The same volume of PBS was injected as a control. Treatment doses were comparable to the sections of the treatment strategies. Mice were dissected and fixed 3 h after NMDA injection. Images of pCRMP2 and NeuN staining indicate that NMDA injection-induced enhancement of CRMP2 phosphorylation at S522 was suppressed by HupA and NAR. Quantitative analysis of RGC with positive CRMP2 phosphorylation signals demonstrated that HupA and NAR inhibited CRMP2 phosphorylation in the retina after 3 h of NMDA injection.

### RGC Degeneration and IRL Thinning Decreased in the NMDA-Administered Model

**Fig. 2:**
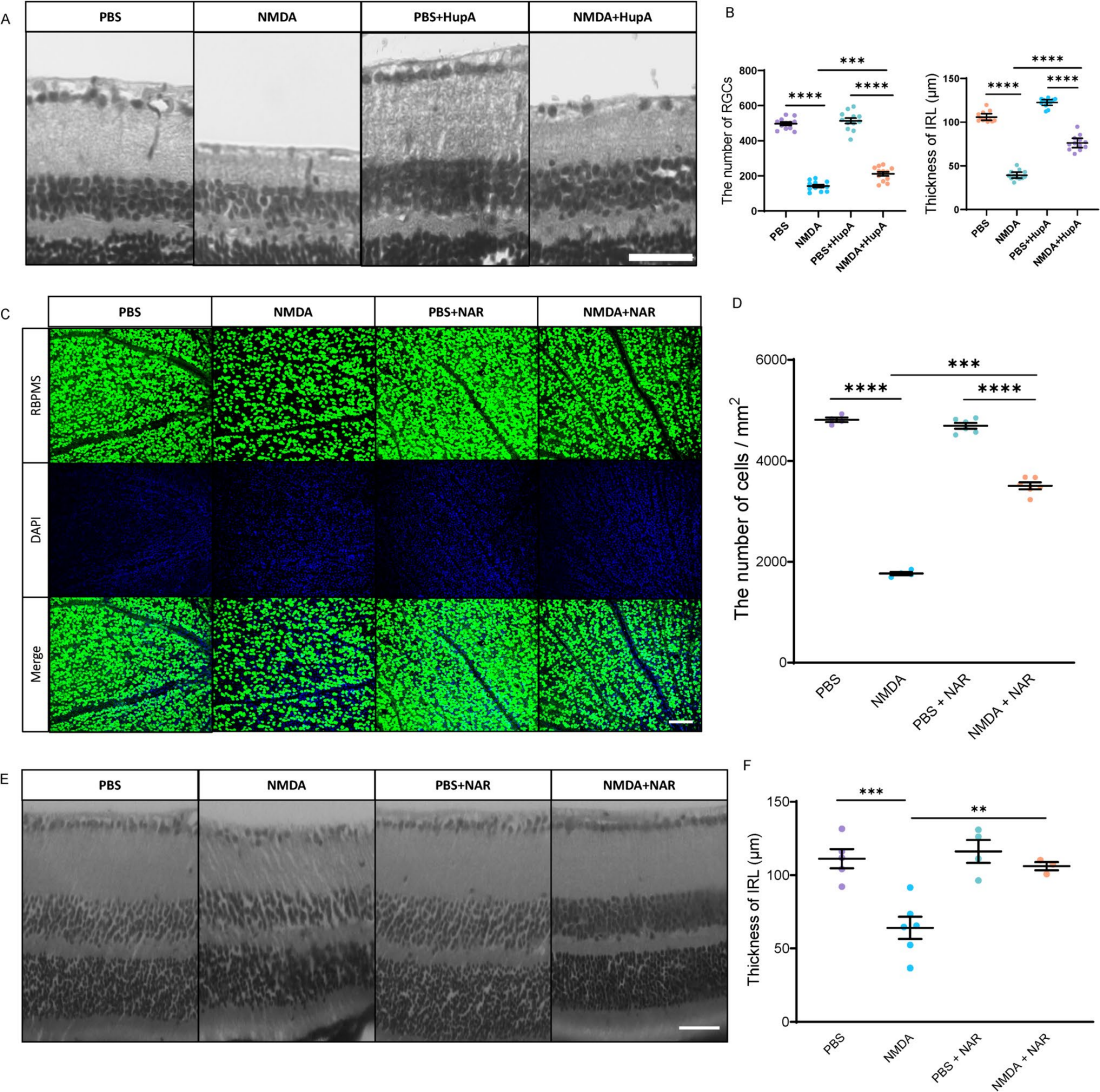
Drug treatments decreased the retinal ganglion cell (RGC) degeneration and inner retinal layer (IRL) thickness reduction in the *N*-methyl-d-aspartate-administered mice. RGC survival and suppression of IRL thickness reduction in retinas were measured after mice were treated with HupA (NMDA + HupA) or NAR (NMDA + NAR) or were untreated (NMDA) for 1 week after NMDA and PBS injection. **A**, **B** H&E staining results from HupA-treated group. **A** Representative images of H&E staining on each side of the retina in PBS, NMDA, PBS + HupA and NMDA + HupA mice. Scale bar, 50 μm. **B** Quantitative analysis of the ratio of the number of RGCs on the NMDA-treated side to that on the PBS-treated side. The thickness of IRL was calculated. (*n* = 6). **C**, **D** Immunostaining of the retina using anti-RBPMS antibody on both NMDA- and PBS-injected sides. **C** Representative immunostaining images on each side of the retina in PBS, NMDA, PBS + NAR and NMDA + NAR mice. Scale bar, 100 μm. **D** The ratio of the number of RGCs on the NMDA-treated side to that on the PBS-treated side was calculated. (*n* = 8). **E**, **F** H&E staining results from NAR-treated group. **E** Representative images of H&E staining on each side of the retina in PBS, NMDA, PBS + NAR and NMDA + NAR mice. Scale bar, 20 μm. **F** The thickness of IRL was calculated. *n* = 6 mice. Data are presented as the mean ± standard error of the mean (SEM) (*n* = 6). **p < 0.01; ***p < 0.001; ****p < 0.0001

We recently revealed that inhibiting CRMP2 phosphorylation in NTG mouse models can be a potential therapeutical strategy for NTG (Brahma et al., 2022). H&E staining was performed to measure the RGC loss and IRL thinning in the NMDA-administered model with HupA and NAR treatment. The wild-type mice were fed HupA and NAR with regular food powder 1 day before NMDA administration. The same volume of PBS was injected as a control. Treatment doses were comparable to the sections of the treatment strategies. Mice were sacrificed 1 week after the injection with continuous oral drug delivery. Immunostaining analysis was performed to examine RGC survival (Fig. 2A, C). RGC degeneration in NMDA-injected mice treated with NAR had a survival effect (Fig. 2B). There was a significant increase in IRL thickness in the NAR-treated group compared to the NMDA-treated group (Fig. 2D). Same measurement standards were analyzed by H&E staining in the HupA-treated group. RGC loss and IRL thickness were reduced in the HupA-treated mice (Fig. 2E, F).

### CRMP2 Phosphorylation was Inhibited by Drug Treatment in the GLAST Mutant Mouse Model

**Fig. 3:**
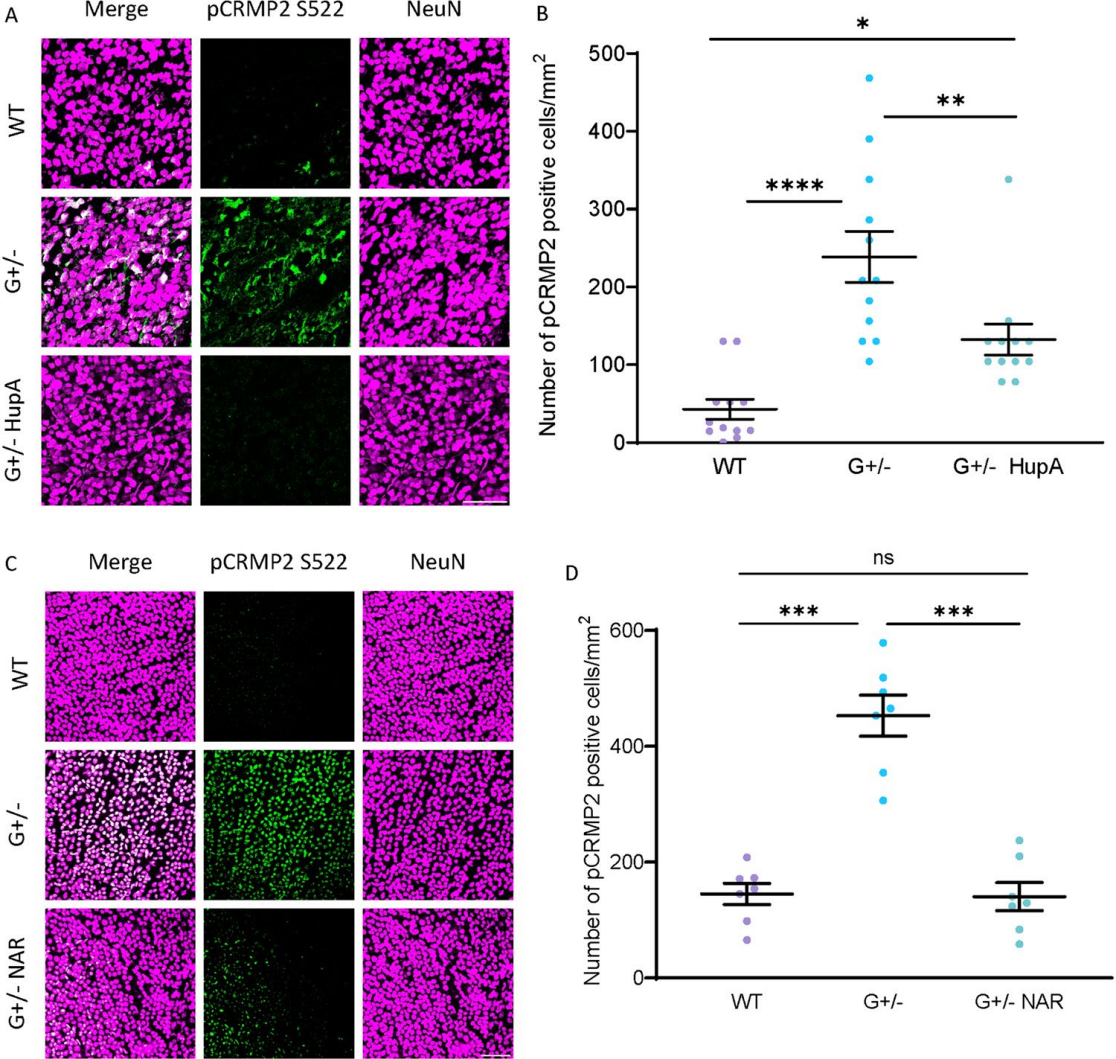
Drug treatments suppressed elevated collapsin response mediator protein-2 (CRMP2) phosphorylation (pCRMP2) at S522 in the retina of GLAST mutant mouse mice of 10-week-old. HupA and NAR suppressed elevated pCRMP2 (S522) in the GLAST+/− mice. The WT mice were used as a control. **A**, **C** Immunostaining images of flat-mount mouse retina were shown. Scale bar, 100 μm. In the retinas of drug-administered mice, lower levels of pCRMP2 (S522) were detected by immunostaining with anti-NeuN and anti-pCRMP2 (S522) antibodies (NeuN: a marker of retinal ganglionic cells). **B**, **D** Quantitative analysis of pCRMP2-positive cells. Data are presented as the mean ± standard error of the mean (SEM) (*n* = 6) for all groups. G+/−, GLAST+/− mice; G+/− + HupA, GLAST+/− mice treated with HupA; G+/− + NAR, GLAST+/− mice treated with NAR; *p < 0.05; ***p < 0.001; ****p < 0.0001; *ns* not significant

Based on previous studies on the GLAST mutant mouse model, RGC degeneration starts as early as when the mice were 3 weeks old and continuously lost until 10 weeks old (Kayama et al., 2011; Sano et al., 2019). In the RGC of 4-week-old GLAST+/− mice, the elevation of CRMP2 phosphorylation at S522 was detected (Brahma et al., 2022). In this study, we apply the same techniques and a 10-week-old mouse model to assess the drug’s effect on reducing CRMP2 phosphorylation at S522 (Fig. 3). The positive signals of CRMP2 phosphorylation in the RGC are represented by the white signals in the merged immunostaining image. Compared with wild-type mice, the retina of 10-week-old GLAST heterozygous (G+/−) mice exhibited substantially higher levels of CRMP2 phosphorylation in the RGC. Compared to the G+/− mice without drug treatment, G+/− mice showed an apparent decrease in CRMP2 phosphorylation positive signals in RGCs when constantly treated by oral administration with HupA and NAR from 3-week-old until the day of dissection at 10-week-old.

### HupA & NAR Suppress RGC Loss in the GLAST Mutant Mouse Model

**Fig. 4:**
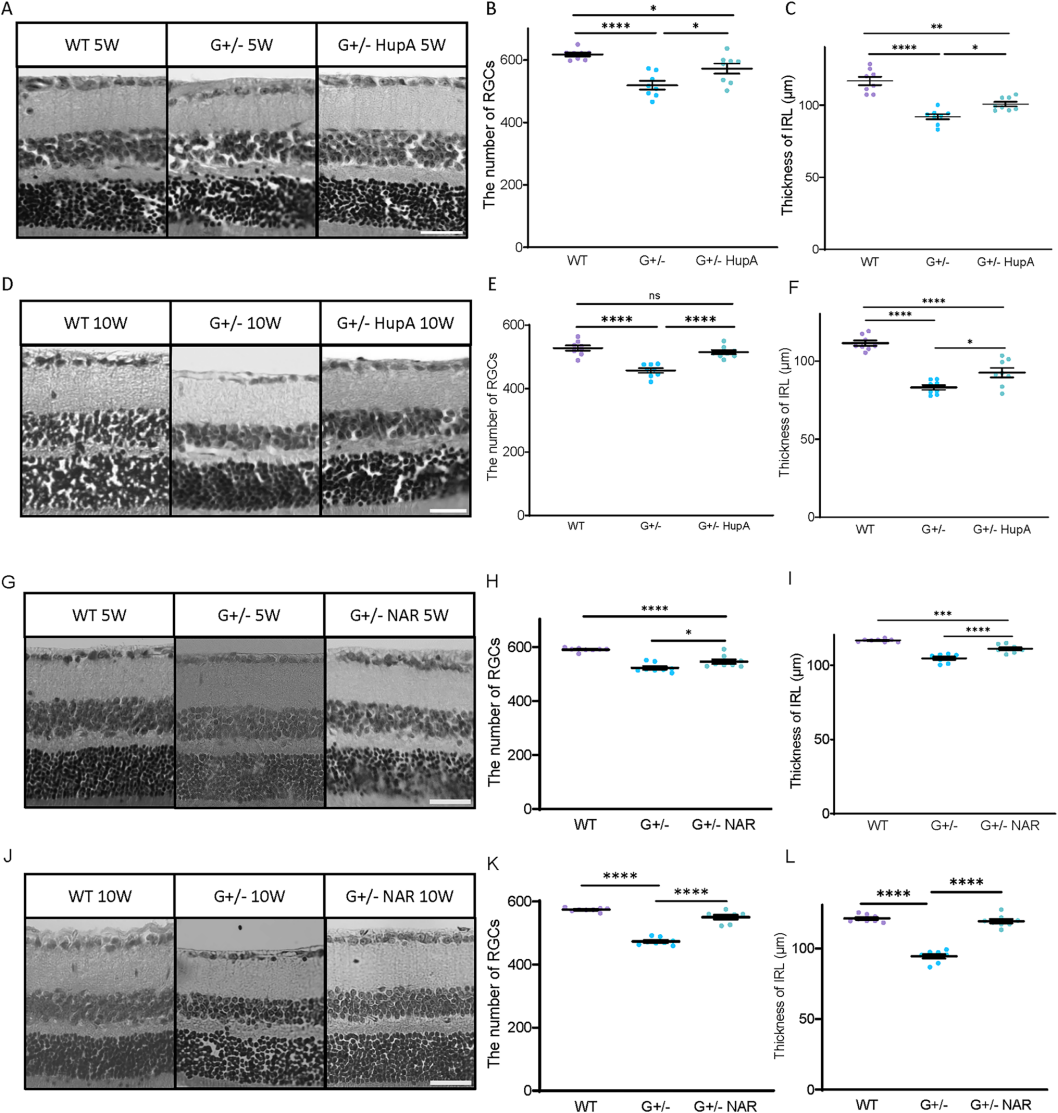
Drug treatments suppressed the retinal ganglion cell (RGC) loss and inner retinal layer (IRL) thickness reduction in 5-week and 10-week GLAST mutant mice. Mice treated with HupA and NAR suppressed RGC death and IRL thickness reduction in 5-week-old and 10-week-old GLAST+/− mice. **A**, **D**, **G**, **J** Representative images of hematoxylin and eosin staining of paraffin-embedded retinal sections. A slower progression in RGC death was observed in the GLAST mutant treated with HupA and NAR from 3 weeks until 10 weeks. **B**, **C**, **E**–**G**, **I**, **K**, **L** Quantitative analyses of the number of cells in the ganglion cell layer (GCL) and IRL thickness, respectively. Figures **B**, **C**, **E**, and **F** show data from 5-week-old mice. Figures **H**, **I**, **K**, and **L** show data from 10-week-old mice. Scale bar, 50 μm. Data are presented as the mean ± SEM, *n* = 8 for 5 weeks old mice, *n* = 6 for 10 weeks old WT mice and *n* = 8 for 10 weeks old G+/− and HupA-treated mice. WT, wild-type; G+/−, GLAST+/− mice; G+/− + NAR, GLAST+/− mice treated with NAR; *p < 0.05; **p < 0.01; ***p < 0.001; ****p < 0.0001; *ns* no significant

To determine whether HupA and NAR can suppress RGC degeneration and IRL thickness, we treated the GLAST mutant mice orally from 3-week-old until 5- and 10-week-old before dissection. Treatment doses were comparable to the sections of the treatment strategies. The GLAST+/− and GLAST−/− mice had considerably fewer RGCs than the wild-type mice (Fig. 4). When comparing the G+/− mice with and without drug treatment, mice treated with HupA for 5 or 10 weeks showed some degree of protection against RGC loss, and mice treated with NAR showed a decrease in RGC loss and alleviation of IRL disintegration.

### HupA & NAR Decrease Oxidative Stress in the GLAST Mutant Mouse Model

**Fig. 5:**
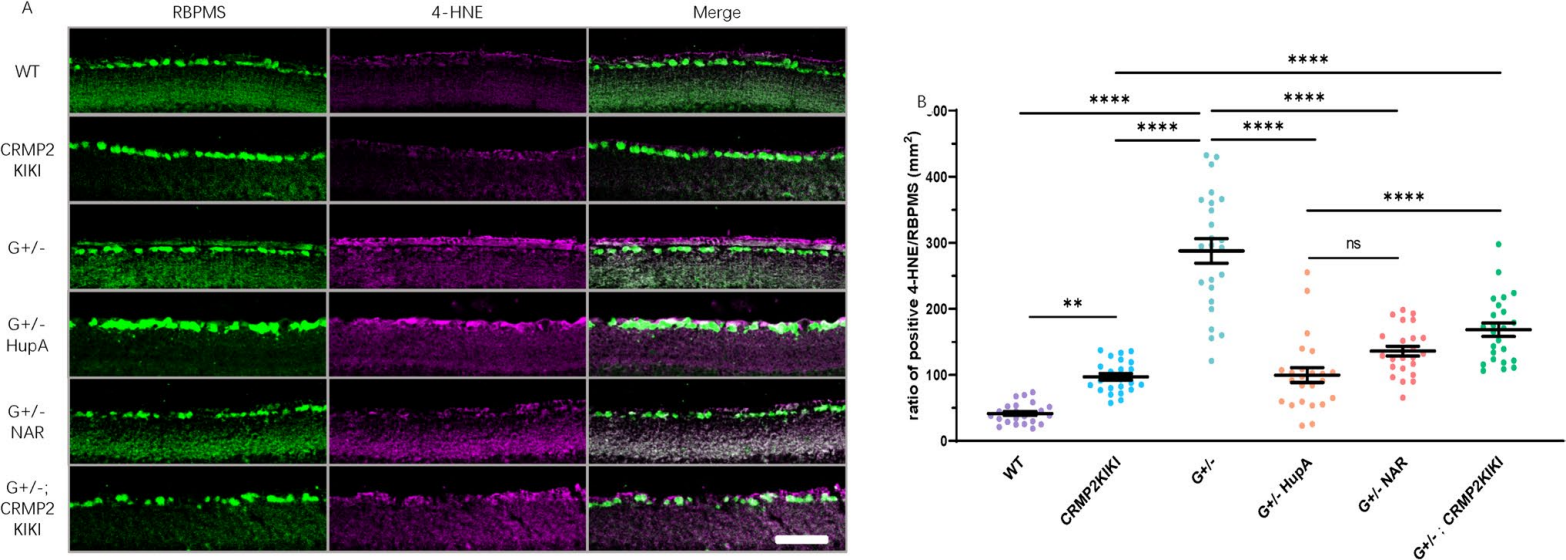
Suppression of oxidative stress by huperzine A and naringenin administration in the retina of GLAST mutant and CRMP2KI/KI mice. HupA and NAR suppressed oxidative stress in a NTG mouse model. **A** Immunostaining images of 4-HNE (a marker of oxidative stress) expression level in the cryosection mouse retina from the wild-type (WT), CRMP2KI/KI (KI/KI), GLAST+/− (G+/−), GLAST+/− + NAR (G+/− + NAR), and GLAST+/−; CRMP2KI/KI (G+/−; KI/KI) mice. **B** Quantitative analyses of the 4-HNE staining in the ganglion cell layer (GCL). Data were normalized to the intensity of 4-HNE staining in the GCL of the WT mice (100%) and are presented as the mean ± SEM (*n* = 6 per group). **p < 0.01; ****p < 0.0001; *ns* no significant. Scale bar, 100 μm

Previous studies on NTG showed the involvement of oxidative stress and glutamate toxicity (Harada et al., 2019). We conducted immunostaining for the oxidative stress marker, 4-HNE, in the retina of GLAST+/− mice (model for NTG), wild-type mice (control), and HupA- and NAR-treated GLAST+/− mice (Fig. 5). Compared to the wild-type and NAR-treated GLAST+/− mice, the GLAST+/− mice had considerably higher levels of 4-HNE immunoreactivity in the RGCs. In the same conditions, we also evaluated oxidative stress in the GLAST+/− : CRMP2KI/KI and CRMP2KI/KI mice. The retinas of GLAST+/− : CRMP2KI/KI mice showed lower levels of oxidative stress than those in the GLAST+/− mice. However, the statistical data showed a higher intensity of 4-HNE immunoreactivity in the RGCs of the GLAST+/− : CRMP2KI/KI mice than in the GLAST+/− mice treated with HupA and NAR. These results suggest that besides suppressing glutamate toxicity in the GLAST mutant and NMDA-injected mice, HupA and NAR inhibited RGC degeneration by reducing oxidative stress in the mouse retina through its CRMP2-dependent and independent effects.

## Discussion

One previous study showed that HupA inhibited GSK3α/β activity and increased the β-catenin level in the brains of transgenic mice (Wang et al., 2011). Studies on New Zealand rabbits demonstrated the retinal neuroprotective and intraocular pressure-lowering effects of HupA (Yu et al., 2021). CRMP2 is phosphorylated by Cdk5, enabling GSK3 to further phosphorylate it (Cole et al., 2006). CRMP2 is phosphorylated by GSK3β and loses its capacity to bind tubulin, a protein essential for microtubule assembly (Uchida et al., 2005). CRMP2 modification is involved in the development of the nervous system and has been implicated in pathological conditions like AD. In a study of the amphetamine-induced hyperlocomotion model, HupA was identified as a non-direct suppressor of GSK3-induced pCRMP2 (Zhao et al., 2020).

NAR has therapeutic effects in many diseases. Some studies have shown similarities in NAR’s effect on diseases, such as AD (Eraslan et al., 2015; Maurano et al., 2018). NAR can bind to CRMP2, reduce phosphorylation at Thr514, and ameliorate memory deficits and AD-like pathology in 5XFAD mice (Yang et al., 2017). NAR might have therapeutic potential for protecting against ocular ischemic diseases because the harmful effects induced by ischemia/reperfusion injury in the rat retina were prevented by inhibiting apoptosis of retinal cells by NAR (Kara et al., 2014).

In this study, we showed that CRMP2 phosphorylation was suppressed by HupA and NAR (Fig. 1). The NTG mouse models, GLAST mutant and NMDA-injected mice, were evaluated after treatment with HupA and NAR. HupA and NAR had a positive effect on both models; RGC degeneration was suppressed, and the reduction in IRL thickness was inhibited after the treatment (Figs. 2, 4). The RGCs of GLAST+/− mice showed an increase in 4-HNE, a marker of oxidative stress (Kikuchi et al., 2020). Accordingly, we treated the mice with HupA and NAR and observed a decrease in 4-HNE in the NAR-treated GLAST+/− mice of 10-week-old (Fig. 5). HupA and NAR had a suppressive effect on the retina of GLAST+/− mice by reducing oxidative stress. When we compared 4-HNE immunoreactivity in the RGCs from the drug-treated GLAST+/− mice with those from the GLAST+/− : CRMP2KI/KI mice, reduction in 4-HNE immunoreactivity in the RGCs was more prominent (Fig. 5), suggesting that HupA and NAR may have effects on CRMP2 phosphorylation suppression and anti-oxidative effects. These findings suggest that HupA and NAR are potential therapeutic options for treating NTG. Furthermore, by comparing the 4-HNE signals of the drug treaded groups and G+/−; CRPM2KIKI groups, it was evident that oxidative stress was significantly lower in the HupA group than in the G+/−; CRPM2KIKI group, whereas in the NAR group it was not significant. Effect of HupA and NAR on RGC protection and CRMP2 phosphorylation inhibition have been proved in this study which gives NTG therapeutical treatment a clue in future. However, the mechanism still not fully elucidated, future studies should also explore the delivery systems, effectiveness, safety, and bioavailability of HupA and NAR in treating NTG.

## Data Availability

The data that support the findings of this study are available on request from the corresponding author.

## Abbreviations

NTG: Normal tension glaucoma
IOP: Intraocular pressure
POAG: Primary open-angle glaucoma
AD: Alzheimer’s disease
CRMP2: Collapsin response mediator protein 2
NAR: Naringenin
KO: Knockout
KI: Knock-in
NMDA: *N*-methyl-d-aspartate
H&E: Haemotoxylin and eosin
PBS: Phosphate buffer saline
IRL: Inner retinal layer
SEM: Standard error of the mean
